# Integrating one health in national health policies of developing countries: India’s lost opportunities

**DOI:** 10.1186/s40249-016-0181-2

**Published:** 2016-10-03

**Authors:** Pranab Chatterjee, Manish Kakkar, Sanjay Chaturvedi

**Affiliations:** 1 Public Health Foundation of India, Plot no 47, Sector 44, Institutional Area, Gurgaon, 122 002 India; 2 Department of Community Medicine, University College of Medical Sciences, Academic, Block, 4th Floor, Dilshad Garden, Delhi 110 095 India

**Keywords:** One Health, Emerging infectious diseases, Health Policy, Zoonoses, Intersectoral coordination, Multidisciplinary, Transdisciplinary

## Abstract

**Background:**

Globally, the threat of infectious diseases, particularly emerging infectious diseases, originating at the human-animal-environment interface, has caught health systems off guard. With forecasts that future pathogen emergence will be centred in hotspots in Asia, Africa, and Latin America, the need to prepare policy frameworks that can combat this threat is urgent.

**Discussion:**

Emergence of diseases such as avian influenza and Ebola virus disease, which threatened social disruption, have established the need for intersectoral coordination/collaboration. These events led to the initiation of establishing institutionalised collaborative frameworks in India to adopt a One Health approach to disease prevention and control. However, the gains made in influenza control could not be adapted to other infectious diseases. Intersectoral coordination was briefly carried out, more as a reactive response to threats. The systemic failure to sustain such efforts have therefore, only undermined a coordinated response. The recent draft National Health Policy, 2015, has also failed to establish the need for intersectoral coordination in disease control approaches. Neglecting the need to endorse linkages between human health, animal health and husbandry, agriculture, and environmental sectors, has led to duplicative and weak response systems.

The absence of health impact assessment with respect to the development agenda in policies, has cast negative effects on the health and wellbeing of man, animal, and the environment. Lack of attention to building core capacity in these critical sectors has further raised challenges in designing and deploying mitigation strategies. With developing countries like India being home to a major portion of the world’s poorest livestock farmers, the absence of a policy discourse that endorses the One Health approach in development and health policies is a major hurdle in eliminating poverty and poverty-related diseases.

**Conclusions:**

The adoption of One Health approaches in health and related sectoral policies is a critical policy requirement for India and other developing countries. The goal should be to not just establish preparedness plans, but also to encourage a policy environment where assessment and mitigation of downstream impacts of different agenda are incorporated.

**Electronic supplementary material:**

The online version of this article (doi:10.1186/s40249-016-0181-2) contains supplementary material, which is available to authorized users.

## Multilingual abstracts

Please see Additional file 1 for translations of the abstracts into the five official working languages of the United Nations.

## Background

The belief that infectious diseases were conquered and the book on communicable diseases was about to be closed has amounted to nothing but hubris. Emerging infectious disease (EID) events of the recent past have seriously damaged these claims over and over again, mostly through diseases that have emerged at the human animal interface. This war between man and microbe might be the inescapable nemesis we have to deal with.

On an average, a new disease has emerged or re-emerged each year since the Second World War; 75 % of these have been from an animal source; [1] between 1940 and 2004, 335 pathogens emerged, with 60 % being of zoonotic origin and 70 % from wildlife [2]. It is forecast that future pathogens will emerge mostly from a wildlife source from the so called “EID hotspots” in tropical Africa, Latin America, and Asia [3]. In fact, of the 1 415 pathogens known to affect humans, 61.6 % are of animal origin [4, 5]. Of the estimated 30 million species existing on planet earth, less than 2 million have been described. Assuming one pathogen associated with each of these species, their pathogenic potential for humans is immense [6]. While the hotspots signify “known” unknowns, the pool of undescribed species constitutes a vast ‘unknown’ unknown. Future threat of infectious diseases is thus not only huge, but also full of uncertainty and should certainly not be undermined.

South Asia has been identified as one of the major hotspots for infectious diseases, with India contributing maximally to the burden of zoonoses, poverty, and reliance on livestock [7]. The emergence of India as a zoonotic hotspot has a wider impact for the region and on global health, raising questions of global preparedness, especially in the context of emerging and re-emerging diseases with epidemic potential.

## Discussion

### Established mechanisms of coordination and missed opportunities

Given the uncertainty surrounding EIDs and their potential to cause explosive outbreaks, the need for a cogent response, characterised by strong multisectoral linkages, and rooted in transdisciplinary approaches, has been felt for a while now [8–13].

The emergence of the H5N1 influenza, and the resulting policy and public panic, led to the conceptualisation of multisectoral linkages in India, with human health, animal health, and wildlife sectors coming together to combat the problem. The collaboration was institutionalised in the form of an Inter-Ministerial Task Force and Joint Monitoring Group at the national level, with coordination mechanisms established all the way down till the district level [14]. Written standard operating procedures (SOPs), in the form of avian influenza contingency plans, were developed and followed in subsequent outbreaks. The protocols ensured successful stamping out of the virus from most locations, though some of the north eastern states are now endemic, with porous international borders playing an important role in the continued transmission [15].

While the avian influenza preparedness and response have been success stories for India, the opportunity created could not be capitalised on. The scope of these coordination mechanisms remain limited and have not been extended to cover zoonoses and wider sets of issues emerging at the human-animal-wildlife interface. Several subsequent zoonotic disease events, occurring nationally and internationally, such as Crimean-Congo Hemorrhagic Fever (CCHF), Ebola Virus Disease (EVD), Middle East Respiratory Syndrome Coronavirus (MERS-CoV), brought the sectors together briefly, culminating into a national programme for intersectoral coordination. A proposal for the same was submitted by the National Centre for Disease Control (NCDC) to the Planning Commission Working Group on the disease burden of communicable diseases for the 12th 5 Year Plan [16].

Central to this proposal was the recruitment of a veterinarian at the Integrated Disease Surveillance Project (IDSP) State Surveillance Unit (SSU) in each of the states. This functionary is proposed to serve as a bridge between the two sectors, though limited evidence has been produced to support this strategy.

Around the same time, the National Standing Committee on Zoonoses (NSCZ) was activated [16]. The Standing Committee is coordinated by the Ministry of Health. Ever since its activation, apart from facilitating limited academic discussions, it has struggled to ensure ownership by other sectors. Also, the policy relevance of its existence has so far remained uncertain.

Similarly, the Indian Council of Medical Research (ICMR), the research arm of the Ministry of Health & Family Welfare (MoHFW), responded by announcing requests for proposals (RFPs) for collaborative research, though it is unclear as to how the disease/s and research priorities had been identified for this research funding. ICMR’s efforts to promote intersectoral collaborative research culminated into discussions around setting up of a Centre for Zoonoses Research, in partnership with the Indian Council of Agricultural Research (ICAR) [17]. Setting up of such an institution will likely lead to duplication of efforts and inefficient utilisation of funds and resources, as preliminary research by our group shows a vast existing capacity for zoonotic disease research in India – over 300 institutions across different sectors/ministries are engaged in cutting edge laboratory and epidemiological research on zoonotic infections (unpublished observations, Manish Kakkar). Given this scenario, mapping and strategic networking of existing capacity could be a more efficient and sustainable solution.

The review of the institutional capacity for research on zoonoses has also shown that a majority of the investments that have gone into building institutional capacity, as well as research output on priority zoonoses from India, have been produced by the human health sector as opposed to the veterinary sector. Thus, it is no surprise that zoonoses research output in India has been found wanting in its policy relevance [18]. A relatively limited focus of the veterinary sector on human diseases of animal origin could be explained in part by competing priorities that are further affected by the limited core capacity of the veterinary and wildlife sectors in India (35 veterinary and 1 wildlife institution versus more than 390 medical colleges) [19]. Moreover, given the likely source of their emergence, the policy disconnect in our preparedness and response to EID threats, as reflected in uneven sectoral investments, is also quite obvious.

Thus, intersectoral mechanisms aimed at operationalising One Health approach based policies appear to be a set of uncoordinated ad-hoc efforts. It is difficult to point out a single factor that has led to this inertia. A multitude of factors may have had contributory effects. EIDs have been perceived as a human health problem, thereby impeding sectorally integrated policies. There is a lack of evidence on operational frameworks, largely driven by a disconnect between research output and policy needs. A mismatch in sectoral capacities, against the backdrop of vastly stretched existing human, animal health and wildlife disease management systems, has resulted in competing priorities and reactive actions in the event of emergencies. This interplay of multiple factors has led to a lack of appreciation of the benefits of the One Health approach, failing to trigger investments, and appropriate effective intersectoral action. However, the biggest missing link has been an overarching One Health policy, or at least a One Health orientation of existing sectoral policies.

### Towards a One health policy for India

Preparing ourselves for the infectious disease challenge of the 21st century would mean that we have to go beyond the eco-epidemiological approaches and address the vast systemic weaknesses in dealing with EIDs through a holistic approach instead. The key to this holistic approach is to establish linkages between the human health, animal health and husbandry, agriculture, and environment sectors. Response in one sector should incorporate impact assessment and mitigate downstream adverse effects on the other sectors as well [20].

Central to this holistic approach should be a policy framework that recognises the EID challenges that India is up against and endorses the need for intersectorality. From such policies should flow operational frameworks that allow partnerships not just across sectors, but also across disciplines. The policy should provide an enabling environment for building core capacity in sectors that play a critical role in responding to EID challenges.

India’s Draft National Health Policy 2015, which was framed in response to the changing healthcare needs of the nation, was the perfect opportunity to leverage systems into breaking out of their water-tight, siloed existence. While the usual process for drafting of health policies in India considers representation of experts from within the sector and interfacing with those from outside, the 2015 draft was unique as it was put in the public domain after drafting (on the website of the Ministry of Health and Family Welfare) and sought comments from all relevant stakeholders. Unfortunately, in spite of being informed by adequate sectoral views and dialogues, the document fails to even mention the terms “zoonoses” and “emerging infectious diseases” [21]. Additionally, the policy was drafted during a time which was a political watershed, and saw a change of regimes at the central government. Naturally, since policy making, at its core, is driven by political interests, it would be naïve to consider that the document would be free from such influence. Unfortunately, the electoral manifesto of the two major political parties that were vying for power at the central level, failed to commit to the control of zoonoses or emerging infectious diseases. This likely apathy could have been reflected in the draft document.

On the other hand, it is interesting to note that there are examples within South Asia that have demonstrated the commitment to deal with the emerging issues following the One Health approach. Bangladesh, for example, has an overarching National Health Policy, and a separate policy framework outlining the Strategic Framework for One Health Approach to Infectious Diseases [22]. The process of developing the National Health Policy is similar in the two countries: being primarily driven by the Ministry of Health and Family Welfare, which constitutes multiple committees to shape the agenda. However, Bangladesh went ahead and developed the strategic One Health framework in 2012, a year after enunciating their National Health Policy, through an inter-ministerial approach, where the Ministries of Health and Family Welfare, Fisheries and Livestock, and Environment and Forests, were brought together on a national One Health platform. This happened through two transdisciplinary workshops to develop a country-level strategy and action plan to operationalise the One Health framework in Bangladesh. The first workshop also incorporated the basics of environmental degradation and agriculture related impacts on health and environment (including food safety issues) within the framework. The framework further identified barriers to operationalisation of a One Health approach to infectious diseases while securing political support from the ministries involved in the policy-framing process.

Globally, there is an impending shift in policy making frameworks as the world is set to migrate from the Millennium Development Goals (MDGs) to the Sustainable Development Goals (SDGs) with the end of the MDG-period on 31st December, 2015. Some observers have expressed their concern about the diffused nature of the health-related targets adopted by the SDGs [23]. However, the SDGs have also emphasised the need to combat neglected tropical diseases and to end outbreaks of EIDs by 2030. It will be interesting to observe whether this policy and funding transition will translate into better appreciation of EID events at the national levels. Also, it is worth observing if this would have a silent, yet substantial impact on the economy and development of nations, as it has been highlighted by the World Bank that six diseases, emerging between 1997 and 2009, caused a cumulative loss of at least US$ 80 billion. According to the World Bank, preventing these outbreaks would have saved an estimated US$ 6.7 billion every year. Notably, these diseases did not emerge as pandemics, and yet placed a significant economic burden on the global community. If they had, indeed, manifested as pandemics, the losses and societal disruption would have been amplified several times over [24]. The impact of these events on small holder farmers in developing countries like India would have been even more serious. In India, nearly 80 % of livestock farmers are small holders; and along with Nigeria, Ethiopia and Bangladesh, India is home to 44 % of the world’s poorest small holding farmers. The disproportionately catastrophic impact of EID events on such vulnerable groups is likely to cause further marginalisation, social disruption, and distress, as was witnessed following the first avian influenza outbreaks in India [7, 25]. Investment in One Health to bolster sectoral convergence and increase systems capacity to combat future EID challenges thus makes economic sense.

## Conclusions

In the absence of a cogent policy environment, India’s approach to dealing with EID events has been reactive at best, and absent, at worst. The lack of an existing framework to guide intersectoral liaison has further impeded responsiveness to EID events, thus contributing to the vulnerabilities, both health and economic. Investments need to be made to establish or re-establish such linkages before cross-sectoral policy dialogues are initiated. In order to reduce redundancy and duplication of policy directives, it is essential that the draft National Health Policy considers elements of transdisciplinarity, which inform mechanisms for establishment of multisectoral approaches to dealing with EID challenges through the One Health approach. It is time that the political and technical leadership in India across sectors recognises the threat from EIDs and responds suitably.

## Additional file

Additional file 1: Multilingual abstracts in the five official working languages of the United Nations. (ZIP 616 kb)

## Abbreviations

CCHF: Crimean-Congo Hemorrhagic Fever
EID: Emerging Infectious Diseases
EVD: Ebola Virus Disease
ICAR: Indian Council of Agricultural Research
ICMR: Indian Council of Medical Research
IDSP: Integrated Disease Surveillance Project
MDG: Millennium Development Goals
MERS-CoV: Middle East Respiratory Syndrome Coronavirus
MoHFW: Ministry of Health and Family Welfare
NCDC: National Centre for Disease Control, Delhi
NSCZ: National Standing Committee on Zoonoses
RFP: Request for Proposal
SDG: Sustainable Development Goals
SOP: Standard Operating Procedures
