# Supplementary material for: Integrating one health in national health policies of developing countries: India’s lost opportunities
Source: Infect Dis Poverty. 2016 Oct 3;5:87. doi: 10.1186/s40249-016-0181-2 (PMC5047123; doi:10.1186/s40249-016-0181-2)
Supplement: Additional file 1: — Multilingual abstracts in the five official working languages of the United Nations. (ZIP 616 kb) [file 40249_2016_181_MOESM1_ESM.zip › Multilingual Abstract.pdf]

Translation of the abstract into the five official working languages of the United Nations

将“同一健康”纳入发展中国家的国家卫生政策：印度失去的机会

Pranab Chatterjee, Manish Kakkar and Sanjay Chaturvedi

摘要：

引言：源于人-动物-环境相互作用的传染病（尤其是新发传染病）打了全球卫生体系一个措手不及。预测亚洲、非洲和拉美将是未来新病原体出现的热点地区，因此迫切需要从政策层面做好迎击准备。

讨论：诸如禽流感和埃博拉等新发传染病对社会的破坏使得需要建立跨部门的协调和合作。这些事件促使印度通过建立制度化的合作框架，以便应用“同一健康”理念来防控这些传染病。但是，在禽流感防控方面的成绩并不适用于其他传染病。跨部门的协调开展的很简单，更多的是对威胁的被动反应。无法持续保持协调只会破坏协调反应。最新的 2015 年国家卫生政策草案也没有将跨部门的协调纳入到疾病防控中。忽视在人、动物和畜牧业、农业和环境部门之间建立联系的需求，将导致应急系统的重复和脆弱。

在政策的发展日程中缺少卫生影响评价将对人、动物和环境的健康造成负面效应。缺乏在这些关键部门建立核心能力的重视使得设计和部署缓解策略面临挑战。像印度这样的发展中国家占了全球最贫困畜牧养殖户的大部分。政策上面缺少对将“同一健康”纳入发展和卫生政策中的支持是消除贫困及贫困相关疾病的重要障碍。

结论：将“同一健康”策略纳入卫生和相关部门的政策中应成为印度及其他发展中国家的关键政策。目标不仅是建立应对方案，而且还应该创建政策环境，以评价对下游不同规划的缓解效果。

Translated from English version into Chinese by Qian Menbao

CHINESE CENTER FOR DISEASE CONTROL AND PREVENTION
NATIONAL INSTITUTE OF PARASITIC DISEASES

Intégrer « Une seule santé » dans les politiques nationales de santé publique des pays en développement : les occasions manquées de l’Inde

Pranab Chatterjee, Manish Kakkar et Sanjay Chaturvedi

Résumé

Contexte : La menace des maladies infectieuses, et en particulier des maladies infectieuses émergentes récemment apparues à l’interface homme-animal-environnement, a pris au dépourvu les systèmes de santé publique dans le monde entier. Les futurs foyers d’émergence de pathogènes apparaîtront principalement en Asie, en Afrique et en Amérique Latine et il est urgent de préparer des cadres stratégiques pour contrer cette menace.

Discussion : L’émergence de maladies comme la grippe aviaire et la fièvre d’Ébola, qui ont failli causer de graves perturbations du tissu social, a mis en évidence la nécessité d’une collaboration et d’une coordination intersectorielles. En Inde, ces événements ont suscité la mise en place de

structures de collaboration institutionnalisées, visant à mettre en place l'approche de prévention et de contrôle des maladies « Une seule santé ». Cependant, les avancées réalisées dans la lutte contre la grippe ne sont pas transposables à d'autres maladies infectieuses. La coordination intersectorielle n'est devenue réalité que brièvement, et plutôt en réaction aux menaces. Ces efforts n'ont pas été repris à l'échelle du système dans son ensemble et n'ont ainsi pas permis de réponse coordonnée. En outre, le récent projet de Politique de santé nationale de 2015 n'affirme pas la nécessité d'une coordination intersectorielle dans la lutte contre les maladies. En négligeant le besoin de prendre en compte les liens entre santé humaine, santé animale et élevage, agriculture et environnement, la planification a créé des systèmes de réponse dupliqués et inefficaces.

L'absence d'évaluation de l'impact sur la santé en relation avec les objectifs de développement des politiques a eu des effets négatifs sur la santé et le bien-être des hommes, des animaux et de l'environnement. Le manque d'efforts pour la constitution des capacités indispensables dans ces secteurs critiques a créé d'autres difficultés encore pour la formulation et le déploiement de stratégies d'atténuation. Alors qu'une forte proportion des éleveurs de bétail les plus pauvres du monde vit en Inde, l'absence d'appropriation de l'approche « Une seule santé » dans les politiques de développement et de santé publique est une obstacle majeur à l'élimination de la pauvreté et des maladies qui lui sont liées.

Conclusions : L'adoption des approches « Une seule santé » dans le domaine de la santé publique et dans les politiques sectorielles connexes est indispensable en Inde comme dans les autres pays en voie de développement. Le but ne doit pas être simplement de rédiger des plans de préparation mais aussi d'encourager un environnement politique dans lequel l'évaluation et la réduction des impacts en aval de différents programmes sont pris en compte.

Translated from English version into French by Suzanne Assenat, through

TRANSLATORS
WITHOUT BORDERS

Введение системы One Health в государственную политику здравоохранения в развивающихся странах: потерянные возможности Индии

Пранаб Чаттерджи, Маниш Каккар и Санже Чатурведи

Тезисы

Историческая справка: В глобальном плане угроза инфекционных заболеваний, в частности возникающие инфекционные заболевания, появляющиеся в среде человек – животное – природа, заставило систему здравоохранения врасплох. Прогнозирование специалистов о том, что следующая вспышка патогенов будет сконцентрирована в сложных местах Азии, Африки и Латинской Америки говорит о том, что необходимость подготовить основы политики для борьбы с этой угрозой остро актуальна

Описание: Возникновение таких заболеваний как птичий грипп и заболевание вируса Эбола, которые угрожали социальной дестабилизацией, выявили необходимость межсекторного

сотрудничества. Эти события привели к предложению создать в Индии институционализированную политику сотрудничества для принятия системы подхода One Health для контроля и предупреждения заболеваний. Однако, полученные результаты при контроле гриппа не могли быть применены к другим инфекционным заболеваниям. Межсекторное сотрудничество было использовано кратковременно и скорее в качестве реактивного ответа на угрозу. Общесистемная неудача при поддержке таких усилий лишь ослабила сплоченный ответ. Недавний черновой вариант законопроекта государственного здравоохранения (2015) также потерпел неудачу при попытке закрепления необходимости межотраслевого сотрудничества в подходах контроля заболеваний. Пренебрежение необходимостью поддержки связей между человеческим здоровьем, животным здоровьем, а также отраслями сельского хозяйства, земледелия и охраны окружающей среды привели к системам дублирования и слабой реакции.

Отсутствие анализа влияния на здоровье с учетом планов развития политических мер вызвало негативные эффекты на на здоровье и благосостояние человека, животного и окружающей среды. Недостаток внимания для создания ключевых способностей в этих критических секторах создал риск при разработке и применении политики минимизации последствий. Когда развивающиеся страны, такие как Индия, являются основными центрами проживания большинства самых бедных в мире животноводческих фермеров, отсутствие политической системы, которая поддерживала бы подход One Health в политике здравоохранения и развития является основным препятствием в сокращении уровня бедности и заболевания, связанных с бедностью.

Выводы: Принятие политики One Health в здравоохранении и связанных с ним секторах, является критическим политическим требованием в Индии и других развивающизся странах. Задачей должно быть не только разработка планов готовности, но и призыв к политической среде, где укоренились практики анализа и минимизации последующих последствий различных программ.

Translated from English version into Russian by Anna Haas, through

TRANSLATORS
WITHOUT BORDERS

La integración del enfoque de «Salud Única» en las políticas sanitarias nacionales de los países en vías de desarrollo: las oportunidades perdidas de la India

Pranab Chatterjee, Manish Kakkar and Sanjay Chaturvedi

Resumen

Antecedentes: A escala mundial, los sistemas sanitarios no estaban preparados para hacer frente a la amenaza de las enfermedades infecciosas, en especial las enfermedades infecciosas de reciente aparición, que se originan entre las personas, los animales y el medio ambiente. Dado el pronóstico

de que la futura aparición de patógenos se centrará en puntos críticos de Asia, África y Latinoamérica, urge preparar un marco político para hacer frente a esta amenaza.
Discusión: A raíz de la aparición de enfermedades como la gripe aviar y la enfermedad del Ébola, las cuales amenazaron con provocar perturbaciones sociales, se ha establecido que es necesario que exista una colaboración y coordinación intersectoriales. En la India, estos episodios llevaron a constituir un marco colaborativo institucionalizado para adoptar el enfoque de «Salud Única» en la prevención y erradicación de las enfermedades. Sin embargo, los beneficios obtenidos en la erradicación de la gripe no se pudieron conseguir con otras enfermedades infecciosas. La coordinación intersectorial se llevó a cabo de manera breve, más bien como una respuesta de reacción a las amenazas. Por tanto, el fracaso generalizado al tratar de mantener estas iniciativas tan solo ha ido en detrimento de la respuesta coordinada. En el reciente borrador de la Política Nacional de Salud (2015) tampoco se ha podido establecer que una coordinación intersectorial en los enfoques para erradicar las enfermedades sea necesaria. Debido a que no se ha concedido la suficiente importancia a los vínculos que existen entre los sectores de la salud humana y animal, la ganadería, la agricultura y el medio ambiente, se han producido estrategias de respuesta redundantes e incoherentes.
La ausencia de una evaluación de las repercusiones en la salud en el contexto del programa de desarrollo de políticas, ha provocado efectos negativos en la salud y bienestar de las personas y animales, y en el medio ambiente. La falta de dedicación a la creación de una capacidad básica en estos sectores clave ha traído más retos durante el diseño y la aplicación de las estrategias de mitigación. En los países en vías de desarrollo, como la India, donde viven la mayoría de los ganaderos más pobres del mundo, la ausencia de un discurso político que avale el enfoque de «Salud Única» en las políticas de salud y desarrollo constituye un importante obstáculo para erradicar la pobreza y las enfermedades asociadas a esta.
Conclusiones: En la India y en otros países en vías de desarrollo, es de importancia crítica adoptar enfoques de «Salud Única» en las políticas sanitarias y sectoriales relacionadas. El objetivo debería ser no solo el de establecer planes de respuesta, sino también el de fomentar un entorno político donde se incorporen la evaluación y la mitigación de las repercusiones posteriores de los diferentes programas.

Translated from English version into Spanish by Mar Jiménez Quesada, through

TRANSLATORS
WITHOUT BORDERS
