# Supplementary material for: Integrating one health in national health policies of developing countries: India’s lost opportunities
Source: Infect Dis Poverty. 2016 Oct 3;5:87. doi: 10.1186/s40249-016-0181-2 (PMC5047123; doi:10.1186/s40249-016-0181-2)
Supplement: Additional file 1: — Multilingual abstracts in the five official working languages of the United Nations. (ZIP 616 kb) [file 40249_2016_181_MOESM1_ESM.zip › Arabic Abstract.pdf]

دمج مبادرة صحة واحدة في السياسات الصحية الوطنية بالبلدان النامية: الفرص الضائعة في الهند

براناب تشاترجي، ومانيش كاكار وسانجاي شاتورفيدي

تلخيص

معلومات عامة: لقد أثار خطر الأمراض المعدية على الصعيد العالمي، لا سيما الأمراض الناشئة المعدية، التي تظهر على السطح البيني للإنسان والحيوان والبيئة، انتباه النظم الصحية. ومن خلال التوقعات بتمركز ظهور مسببات الأمراض في المستقبل في المناطق الساخنة في آسيا وأفريقيا وأمريكا اللاتينية، فثمة حاجة لإعداد أطر للسياسات التي يمكن من خلالها مكافحة هذا التهديد العاجل.

المناقشة: لقد أثار ظهور أمراض مثل إنفلونزا الطيور ومرض فيروس الإيبولا الذي هدد التمزق الاجتماعي، ضرورة التنسيق بين القطاعات والتعاون فيما بينها. وأدت هذه الأحداث إلى الشروع في وضع أطر تعاونية مؤسسية في الهند لاعتماد نهج مبادرة صحة واحدة للوقاية من الأمراض ومكافحتها. ومع ذلك، فلا يمكن أن تتكيف المكاسب التي تحققت في مكافحة الإنفلونزا مع الأمراض المعدية الأخرى. وقد جرى التنسيق بين القطاعات بإيجاز، وهو بمثابة أكبر استجابة للتهديدات. ولم يقوض الفشل الشامل للحفاظ على هذه الجهود بالتالي إلا القيام باستجابة منسقة. ولم تحقق مسودة مشروع السياسة الوطنية للصحة مؤخرًا، في عام 2015، نجاحًا فيما يتعلق بضرورة التنسيق بين القطاعات في نهج مكافحة الأمراض. إن إغفال الحاجة إلى تأييد الروابط بين صحة الإنسان والحيوان وتربية الحيوانات والزراعة والقطاعات البيئية، أدى إلى ظهور نظم الاستجابة المزدوجة والضعيفة.

وقد ألقى غياب تقييم الأثر الصحي فيما يتعلق بجدول أعمال التنمية في السياسات آثار سلبية على صحة الإنسان والحيوان والبيئة وسلامتهم. وأثار عدم الاهتمام ببناء القدرات الأساسية في هذه القطاعات الحيوية مزيد من التحديات في مجال وضع إستراتيجيات تخفيف الآثار ونشرها. ومع كون البلدان النامية مثل الهند موطن لجزء كبير من أفقر مربي الماشية في العالم، فإن غياب الخطاب السياسي الذي يؤيد نهج مبادرة صحة واحدة في سياسات التنمية والصحة لهو عقبة كبيرة في القضاء على الفقر والأمراض المرتبطة به.

الاستنتاجات: إن إقرار نهج مبادرة صحة واحدة في الصحة والسياسات القطاعية ذات الصلة لهو متطلب السياسة الحاسمة بالنسبة للهند والدول النامية الأخرى. ينبغي ألا يكون الهدف مجرد وضع خطط للتأهب، بل أيضًا تشجيع بيئة السياسات التي تدمج فيها تقييم الآثار السلبية وتخفيفها بالنسبة لجدول الأعمال المختلف.

Translated from English version into Arabic by Fathia Sobhi, through

TRANSLATORS
WITHOUT BORDERS
